# Herbal Remedies for Coccidiosis Control: A Review of Plants, Compounds, and Anticoccidial Actions

**DOI:** 10.1155/2016/2657981

**Published:** 2016-06-20

**Authors:** Thangarasu Muthamilselvan, Tien-Fen Kuo, Yueh-Chen Wu, Wen-Chin Yang

**Affiliations:** ^1^Agricultural Biotechnology Research Center, Academia Sinica, Taipei 115, Taiwan; ^2^Institute of Pharmacology, National Yang-Ming University, Taipei 112, Taiwan; ^3^Department of Life Sciences, National Chung Hsing University, Taichung 402, Taiwan; ^4^Institute of Biotechnology, National Taiwan University, Taipei 106, Taiwan

## Abstract

Coccidiosis is the bane of the poultry industry causing considerable economic loss.* Eimeria* species are known as protozoan parasites to cause morbidity and death in poultry. In addition to anticoccidial chemicals and vaccines, natural products are emerging as an alternative and complementary way to control avian coccidiosis. In this review, we update recent advances in the use of anticoccidial phytoextracts and phytocompounds, which cover 32 plants and 40 phytocompounds, following a database search in PubMed, Web of Science, and Google Scholar. Four plant products commercially available for coccidiosis are included and discussed. We also highlight the chemical and biological properties of the plants and compounds as related to coccidiosis control. Emphasis is placed on the modes of action of the anticoccidial plants and compounds such as interference with the life cycle of* Eimeria*, regulation of host immunity to* Eimeria*, growth regulation of gut bacteria, and/or multiple mechanisms. Biological actions, mechanisms, and prophylactic/therapeutic potential of the compounds and extracts of plant origin in coccidiosis are summarized and discussed.

## 1. Introduction

Each year, over 50 billion chickens are raised as a source of meat, accounting for over one-third of protein food for humans [1]. However, poultry production is often confronted by avian coccidiosis, flu, and other infectious diseases [1]. Avian coccidiosis is characterized as an infectious protozoan disease caused by gut parasites of the genus* Eimeria* (Coccidia subclass) [2]. So far, nine* Eimeria* species,* E. acervulina*,* E. brunetti*,* E. maxima*,* E. necatrix*,* E. praecox*,* E. mitis*,* E. tenella*,* E. mivati*, and* E. hagani*, have been identified from chickens [3]. These parasites can infect and multiply within the mucosal epithelia in different parts of bird guts via oral route. As a result, they cause gut damage (i.e., inflammation, hemorrhage, diarrhea, etc.), morbidity, and mortality in poultry [4]. This disease annually causes a global loss of over 2.4 billion US dollars in the poultry industry, including poor growth performance, replacement of chicks, and medication [1]. Current approaches to constrain avian coccidiosis include anticoccidial chemicals, vaccines, and natural products. Anticoccidial chemicals, coccidiocides, coccidiostats, and ionophores, have long been used as a mainstream strategy to control avian coccidiosis in modern poultry production [5]. Although this strategy is cost-effective and successful, the presence of drug resistance and public demands for residue-free meat has encouraged development of alternative control strategies [5]. Moreover, in European countries, the prophylactic use of anticoccidial chemicals as feed additives has been strictly limited since 2006 and a full ban has been proposed to be effective in 2021 (Council Directive of 2011/50/EU of the European Council). To cope with this global situation, vaccination, composed of one or more strains of wild-type or attenuated* Eimeria* species, is successfully developed as another approach to prevent coccidiosis though their cross-species protection and efficacy may need to be improved. Natural products are emerging as an attractive way to combat coccidiosis. Currently, there are at least four plant products commercially available on the market and they can be used as anticoccidial feed additives in chickens and/or other animals, including Cocci-Guard (DPI Global, USA) [6], a mixture of* Quercus infectoria*,* Rhus chinensis*, and* Terminalia chebula* (Kemin Industries, USA) [7], Apacox (GreenVet, Italy) [8], and BP formulation made up of* Bidens pilosa* and other plants (Ta-Fong Inc., Taiwan) [9]. Besides, investigation of the compounds and/or their derivatives present in anticoccidial plants may inspire the research and development of anticoccidial chemicals. One successful example is halofuginone, a synthetic halogenated derivative of febrifugine, which was initially identified from the antimalarial plant, Chang Shan (*Dichroa febrifuga*) [10]. Despite the aforesaid merits of natural products, several challenges in the anticoccidial use of natural products such as anticoccidial efficacy, identification of active compounds, mechanism, safety, and cost-effectiveness of plant extracts and compounds need to be overcome prior to further applications.

Several reviews were published on the plant extracts and their phytochemicals for avian coccidiosis [1, 11, 12]. To complement and update recent progress on the anticoccidial properties of plant extracts and/or compounds, here we have updated the plants and compounds related to anticoccidial activity, chemistry, and prophylactic/therapeutic potential. New views on the anticoccidial action of plants and compounds are also discussed. Common terms and their definitions used in this paper are presented as follows.

*Anticoccidial Chemicals*. These are chemicals which kill coccidia (coccidiocides) or slow their growth (coccidiostats). Ionophores are a class of polyether chemicals which interfere with ion transport, leading to the death of coccidia. 

*Phytoextract/Plant Extract*. They are a collection of plant ingredients obtained by means of solvent extraction. 

*Phytocompound/Phytochemical*. This refers to any chemical synthesized by plants. 

*Natural Products*. These are compounds/chemicals found in nature including plants, animals, and minerals. 

*Coccidia*. It is a subclass of microscopic, spore-forming, single-celled protozoan parasites belonging to the apicomplexan class.

*Eimeria*. This is a genus of apicomplexan parasites that include various species capable of causing gut disease in poultry and other animals.

### 1.1. Life Cycle of* Eimeria* Species

**Figure 1:**
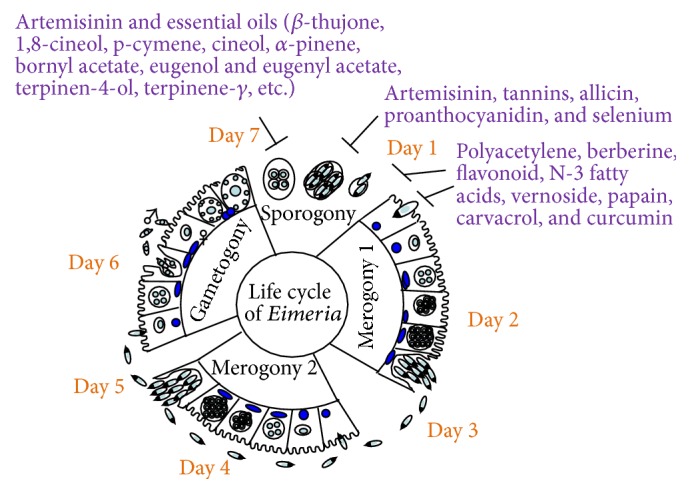
Plant compounds target different stages of the life cycle of* Eimeria* species.* Eimeria* species take 4–7 days to complete their life cycles. They have 3 different developmental stages in poultry: sporogony, merogony, and gametogony. This scheme is modified from the previous publication [40]. Different phytocompounds inhibit the growth of* Eimeria* species at sporogony and merogony stages.

As illustrated in Figure 1,* Eimeria* species have a complex life cycle, consisting of three developmental stages (sporogony, schizogony/merogony, and gametogony). Oocysts excreted in poultry feces sporulate in a favourable environment with high humidity at 25–30°C. Once sporulating oocysts are ingested by birds, physical and chemical agents in their digestive tracts are released and mature infectious sporozoites form sporocysts. The sporozoites enter epithelial cells in the gut, depending on the specific* Eimeria* species, and form trophozoites and, later, schizonts during schizogony/merogony [13]. Merozoites, released from the schizont, can penetrate into the epithelia and continue this merogony stage 2 to 3 times in order to increase the cell number of merozoites at the asexual stage of reproduction. Alternatively, merozoites may enter the sexual stage of reproduction by forming male microgametes, the equivalent of sperm, and female macrogametes, the equivalent of oocytes, in host cells. Following fertilization, the zygotes develop into oocysts and are excreted into poultry stool.* Eimeria* species may require 4–7 days to complete their entire life cycles [14]. Thus, the asexual and sexual stages of reproduction in the* Eimeria* life cycle have been targeted by anticoccidial compounds [4].

### 1.2. Prophylaxis and Therapy for Avian Coccidiosis

Coccidiosis is one of the major problems in intensive poultry farming [1]. In contrast to anticoccidial chemicals and vaccines, the use of medicinal plants and phytocompounds as natural remedies for avian coccidiosis has become an alternative strategy that is easily embraced by eco- and health-conscious consumers. Of course, this strategy is also in compliance with the “anticoccidial chemical-free” regulation enacted by the European Union (Council Directive of 2011/50/EU). Plants produce a broad-spectrum variety of phytochemicals such as phenolics, polyacetylenes, alkaloids, polysaccharides, terpenoids, and essential oils with a large number of bioactivities [12, 15, 16]. Different reports on the use of medicinal plants for diseases suggest that they have the potential to “kill several birds with one stone” because they contain multiple phytochemicals and can intervene in multiple disease-related signalling pathways [17].

## 2. Plants and Compounds for Avian Coccidiosis

Over 300,000 species of flowering plants have been recorded worldwide. So far, less than 1% of them have been explored for use against protozoan diseases [18]. In this section, a total of 68 plants and phytocompounds, which were scientifically tested for suppression of* Eimeria* species, are described and discussed [1, 9, 11, 12].  Table 1 lists 32 anticoccidial plants whose active compounds and modes of action need to be elucidated:* Sophora flavescens* (Fabaceae),* Sinomenium acutum* (Menispermaceae),* Quisqualis indica* (Combretaceae),* Pulsatilla koreana* (Ranunculaceae),* Ulmus macrocarpa* (Ulmaceae),* Artemisia asiatica* (Asteraceae),* Gleditsia japonica* (Fabaceae),* Melia azedarach* (Meliaceae),* Piper nigrum* (Piperaceae),* Urtica dioica* (Urticaceae),* Artemisia sieberi* (Asteraceae),* Lepidium sativum* (Brassicaceae),* Foeniculum vulgare* (Apiaceae),* Morinda lucida* (Rubiaceae),* Commiphora swynnertonii* (Burseraceae),* Moringa oleifera* (Moringaceae),* Origanum* spp. (Lamiaceae),* Laurus nobilis* (Lauraceae),* Lavandula stoechas* (Lamiaceae),* Musa paradisiaca* (Musaceae),* Moringa stenopetala* (Moringaceae),* Solanum nigrum* (Solanaceae),* Mentha arvensis* (Lamiaceae),* Moringa indica* (Moringaceae),* Melia azadirachta* (Meliaceae),* Tulbaghia violacea* (Amaryllidaceae),* Vitis vinifera* (Vitaceae),* Artemisia afra* (Asteraceae),* Quercus infectoria* (Fagaceae),* Rhus chinensis* (Anacardiaceae), and* Terminalia chebula* (Combretaceae). Details about active phytocompounds present in the other anticoccidial plants and their mechanisms are summarized in Tables 2 [Table tab3]–4.

### 2.1. Plants and Compounds That Inhibit the Life Cycle of* Eimeria*

In this section, the phytochemicals and plants, which suppress coccidiosis via intervention with the developmental stages of life cycle in* Eimeria* species in poultry, are discussed. They are summarized in Table 2. Their chemistry and mechanism of action of phytochemicals and plants are also described below and summarized in Table 2 and Figure 1.

#### 2.1.1. *Artemisia annua* and Artemisinin

*A. annua* and its constituent active compound artemisinin have been reported to have anticoccidial action (Table 2). Mechanistic studies show that this compound generated reactive oxygen species (ROS) via degradation of iron-implicated peroxide complex and, therefore, induced oxidative stress. Of note, ROS was documented to directly inhibit sporulation and cell wall formation in* Eimeria* species, leading to interference with the life cycle of* Eimeria* [19–23]. In addition,* A. annua* has lots of phytochemicals, flavonoids, and phenolic compounds which can help birds maintain commensal microflora and take up large amounts of nitrogen. Commensal bacteria play a significant role in enhancing digestion of food and absorption of nutrients and improve innate and acquired immune response in poultry [24].

#### 2.1.2. Condensed Tannins and Pine Bark

The extract from the bark of the pine tree (*Pinus radiata*), which is rich in condensed tannins, was reported to inhibit the life cycle of Coccidia as evidenced by decreased sporulation of the oocysts of* E. tenella*,* E. maxima*, and* E. acervulina* [25]. The mode of action of condensed tannins was suspected to be penetration of the wall of the oocyst and damage to the cytoplasm since the tannins could inactivate endogenous enzymes responsible for the sporulation process. This was further supported by the appearance of abnormal sporocysts in oocysts [25].

#### 2.1.3. Garlic (*Allium sativum*) and Allicin

Garlic and its sulfur compounds, allicin, alliin, ajoene, diallyl sulfide, dithiin, and allylcysteine, are reported to have broad antimicrobial activities which can eliminate negative factors of microbial infections. An* in vitro* study has shown that allicin inhibits sporulation of* E. tenella* effectively [26–28]. The anticoccidial mechanism of garlic and its sulfur compounds remains elusive.

#### 2.1.4. Selenium, Phenolics, and Green Tea (*Camellia sinensis*)

Green tea extracts have been shown to significantly inhibit the sporulation process of coccidian oocysts [29, 30]. Accordingly, the selenium and polyphenolic compounds in green tea are thought to be active compounds to inactivate the enzymes responsible for coccidian sporulation [29, 30].

#### 2.1.5. N-3 Fatty Acids, Flavonoids, Vernoside, and Their Plant Sources

Upon the invasion of* Eimeria* sporozoites into the intestinal epithelium, reactive nitrogen species (RNS) and reactive oxygen species (ROS) are often produced by host cells, leading to the death of sporulating oocysts [21]. Similarly, another study demonstrated that the extracts from* Berberis lyceum*, in which berberine was enriched, inhibited the sporozoites of* E. tenella* in chickens via induction of oxidative stress. Other studies indicated that extracts from* Linum usitatissimum* [21],* Ageratum conyzoides* [31], and* Vernonia amygdalina* [32] controlled coccidian infection via induction of oxidative stress. Moreover, N-3 fatty acids, flavonoids, and vernoside were identified as active compounds present in* L. usitatissimum*,* A. conyzoides*, and* V. amygdalina*, respectively. These compounds were shown to elicit oxidative stress (Table 2). Oxidative stress is known to cause imbalance of oxidant or antioxidant species in the host and is often observed in a wide range of microbial and parasitic infections including coccidiosis [19]. Moreover, these natural extracts not only enhanced chicken growth but also had no noticeable toxicity.

#### 2.1.6. *Carica papaya* and Papain

Two studies have reported that extracts from* C. papaya* leaves significantly inhibit coccidiosis [32, 33]. Little is known about the anticoccidial mechanism. Proteolytic destruction of* Eimeria* by papain and/or inflammatory suppression by vitamin A were proposed as possible mechanisms by which* C. papaya* and its active compounds acted to suppress coccidiosis [32, 33].

#### 2.1.7. Saponin, Betaine, and Their Plant Sources

Hassan et al. demonstrated that dietary supplementation of guar bean (*Cyamopsis tetragonoloba*) suppressed coccidiosis in chickens [34]. This suppression was proposed to be achieved by saponins, presumably the active compounds, which bind with sterol molecules present on the cell membrane of the parasites [34]. Another study also reported that the extracts of* M. cordifolia*,* M. citrifolia*, and* M. arboreus* showed anticoccidial effects in chickens [35]. Saponins were presumed to be the active compounds which could lyse oocysts. In contrast, another report described betaine, an active compound isolated from beet or other plants, as contributing to the stabilization and protection of the epithelial cells in which* Eimeria* multiply [36].

#### 2.1.8. Essential Oils and Their Plant Sources

Essential oils derived from plants showed inhibition of* Eimeria* species at different developmental stages (Figure 1). Essential oils are an important natural product resource, which are rich in many phytocompounds. Both* in vitro* and* in vivo* studies reported that essential oils can be used as feed additives in chickens to control coccidiosis [1, 11, 12]. Bioactive compounds present in the essential oils extracted from* Oreganum compactum*,* A. absinthium*,* Rosmarinus officinalis*,* Anredera cordifolia*,* Morinda citrifolia*,* Malvaviscus arboreus*,* Syzygium aromaticum*,* Melaleuca alternifolia*,* Citrus sinensis*, and* Thymus vulgaris* were able to destroy the parasites, including oocysts and sporozoites (Table 1).

#### 2.1.9. Maslinic Acid

Maslinic acid, an active compound in the leaves and fruit of the olive tree (*Olea europaea*), was originally identified as a novel anticoccidial compound as indicated by the lesion index, the oocyst index, and the anticoccidial index [37]. However, its anticoccidial activity remains unknown.

#### 2.1.10. Proanthocyanidin and Grape Seed

Proanthocyanidin is a naturally occurring polyphenolic antioxidant widely distributed in grape seed and other sources. Grape seed proanthocyanidin extract was shown to reduce* E. tenella* infection as shown by gut pathology, body weight, and mortality [38]. Accordingly, this extract decreased nitric oxide but increased superoxide dismutases in the plasma of chickens [38]. These data suggest that proanthocyanidin from grape seed diminishes coccidiosis via downregulation of oxidative stress.

#### 2.1.11. *Dichroa febrifuga*

*D. febrifuga*, also known as Chang Shan, is a Chinese medicinal herb for protozoan diseases. Zhang and coworkers showed that crude extract of* D. febrifuga* was effective against* E. tenella* infection in chickens [39]. Febrifugine, an alkaloid, was isolated from this plant and its halogenated derivative, halofuginone, was developed as anticoccidial chemical [10].

### 2.2. Plants and Compounds That Modulate Host Immunity against* Eimeria*

**Figure 2:**
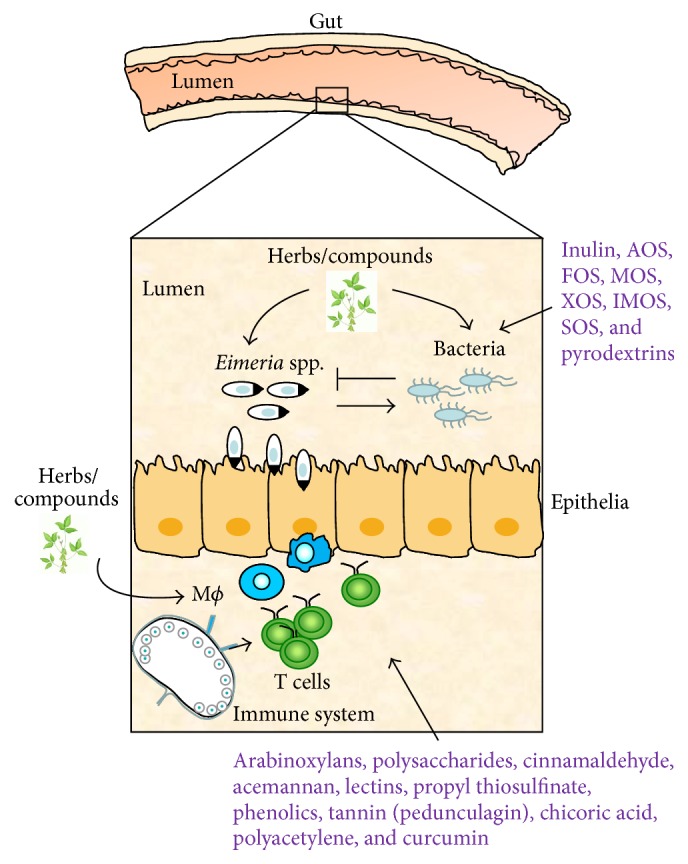
Immune and prebiotic modulation underlying anticoccidial compounds. In the lumens of bird guts, bacteria and* Eimeria* species interact with each other. Particularly, some beneficial bacteria can reduce gut lesions caused by* Eimeria* species. Gut-associated T cells, macrophages, and other immune cells can mount immune responses to harmful* Eimeria* and bacteria. Phytocompounds from plants can inhibit the multiplication of* Eimeria*, expand the growth of beneficial bacteria, and/or boost immunity, leading to controlling* Eimeria* infection in the gut of poultry.

From an evolutionary point of view, birds have a complete immune system consisting of innate and adaptive immune responses [96]. Both immune responses are responsible for coccidial clearance and vaccine immunization [12, 97]. Medicinal plants often have immunomodulatory compounds which boost antimicrobial immune responses to uphold homeostasis of poultry health [91, 98]. Therefore, immunoregulatory plant extracts and compounds could be utilized as an alternative method to reinforce immune response against avian coccidiosis. Immunoregulatory phytochemicals for avian coccidiosis are described in Table 3 and Figure 2.

#### 2.2.1. Arabinoxylans, Wheat (*Triticum aestivum*), and Sugar Cane (*Saccharum officinarum*)

Akhtar and colleagues showed that arabinoxylan, a bioactive compound from wheat bran, improved coccidiosis in chickens as indicated by body weight, oocyst count, and gut lesions [69]. In contrast, Awais and Akhtar reported that different extracts of sugar cane juice and bagasse protected against coccidiosis in chickens as shown by body weight gain, oocyst shedding, lesion score, and anticoccidial indices [99]. The data from both studies revealed that wheat bran arabinoxylan and sugar cane conferred host protection against* Eimeria* infection via natural and adaptive immune response. Cell-mediated immunity seemed to be a key factor in response to coccidiosis in chickens when compared to humoral immunity [69, 99].

#### 2.2.2. Polysaccharides from* Astragalus membranaceus Radix*,* Carthamus tinctorius*,* Lentinus edodes*, and* Tremella fuciformis*

Guo and colleagues reported that the polysaccharides derived from the herb* A. membranaceus Radix* and the mushrooms* L. edodes* and* T. fuciformis* effectively controlled* E. tenella* infection in chickens [80]. Concurrent with the anticoccidial protection, the polysaccharides could enhance anticoccidial antibodies and antigen-specific cell proliferation in splenocytes via cellular and humoral immunity to* E. tenella* in chickens [80]. Their mechanism appeared to stimulate cell proliferation of the lymphocytes via regulation of DNA polymerase activity.

#### 2.2.3. Cinnamaldehyde, Carvacrol,* Capsicum* Oleoresin, and Turmeric Oleoresin

Two phytonutrient mixtures, VAC (carvacrol, cinnamaldehyde, and* Capsicum* oleoresin) and MC (*Capsicum* oleoresin and turmeric oleoresin), were tested for coccidiosis in chickens [70]. The data proved that both combination treatments effectively protected against* E. tenella* infection. Moreover, both treatments exhibited an increase in NK cells, macrophages, CD4^+^ T cells, CD8^+^ T cells, and their cytokines (IFN-*γ* and IL-6) and a decrease in TNFSF15 and IL-17F, leading to induction and elevation of host immunity to kill* E. tenella* in chickens [70].

#### 2.2.4. Aloe and Acemannan

Gadzirayi et al. showed that* A. excelsa* possesses anticoccidial activity in chickens [36, 43, 100, 101] despite lack of information about its mode of action and its active compound(s). Another study stated that aqueous and ethanolic extracts from* A. vera* mounted a cell-mediated immune response as well as a humoral response against coccidiosis in chickens. The immunomodulatory compounds could include aloe polysaccharide acemannan (Figure 2), which binds the mannose receptor on macrophages, stimulating them to produce inflammatory cytokines such as IL-1 through IL-6 and TNF-*α* and eventually suppress coccidiosis as shown by greater weight gain and lower fecal oocyst counts [72, 100].

#### 2.2.5. Oriental Plum (*Prunus salicina*) and Phenolics

One report showed that dietary supplementation of plum fruit powder, rich in phenolic compounds, added to chicken feed significantly diminished* E. acervulina* infection in chickens as demonstrated by increased body weight gain and reduced fecal oocyst shedding [81]. Accordingly, plum fruit powder greatly augmented the transcription of IFN-*γ* and IL-15 and splenocyte proliferation, indicating that plum fruit can boost immune response to coccidiosis.

#### 2.2.6. Mushroom (*Fomitella fraxinea*) and Lectin

One study showed that the lectin derived from a mushroom,* F. fraxinea*, protected chickens from* Eimeria* challenge via enhancement of both cellular and humoral immune responses [74]. This work also suggested that this mushroom could enhance both immune responses to* Eimeria* species in chickens. This study implies that immunoregulatory botanicals such as mushroom can improve poultry growth and development via immune protection from infectious pathogens and toxins. Moreover, botanicals, containing micro- and macronutrients, can increase growth performance in poultry.

#### 2.2.7. Propyl Thiosulfinate and Propyl Thiosulfinate Oxide

One study showed that garlic compounds, propyl thiosulfinate (PTS) and propyl thiosulfinate oxide (PTSO), could alter the expression levels of 1,227 transcripts related to intestinal intraepithelial lymphocytes (IEL) in chickens. PTSO/PTS was shown to activate transcription factor, NF-*κ*B, which plays a key role in regulating the immune response upon infection. Therefore, it seems that a combination of PTSO and PTS rendered chickens more resistant to experimental* E. acervulina* infection and augmented adaptive immunity, including a higher antibody response and greater splenocyte proliferation, compared with control chickens [75]. Another* in vitro* study showed that PTS could stimulate splenocyte proliferation and directly kill the sporozoites, pointing to the same conclusion [75].

#### 2.2.8. Tannins and Chicoric Acid from* Emblica officinalis* and* Echinacea purpurea*

Tannins and chicoric acid, isolated from* E. officinalis* [77] and* E. purpurea* [102], respectively, were reported to effectively elicit humoral immune response against coccidial infection in chickens. However, the mechanism by which both compounds boost anticoccidial immunity is not clear.

### 2.3. Plants and Compounds That Possess Prebiotic Properties

Like in humans, gut microbiota are important for health and disease in poultry [103]. Gut microbiota perform multiple functions involved in nutrient digestion, gut development and growth, establishment/maintenance of the immune system, suppression of pathogenic microbes, microbial infections, and so forth [8, 104–109]. Thus, promotion of beneficial microbes and reduction of harmful microbes contribute to growth performance and health in poultry [103]. Several studies have indicated that probiotics, containing one or multiple species of* Lactobacillus*,* Enterococcus*, and/or* Bifidobacterium*, can reduce coccidiosis and enhance growth performance in chickens [57, 97, 104, 110, 111]. Currently, little is known about the anticoccidial mechanisms of probiotics. These modes of action have been proposed: maintaining a healthy balance of bacteria by competitive exclusion and antagonism, promoting gut maturation and integrity, modulating immunity and preventing inflammation, altering metabolism by increasing digestive enzyme activity and decreasing bacterial enzyme activity and ammonia production, improving feed intake and digestion, and neutralizing enterotoxins and stimulating the immune system [91, 108]. On the other hand, coccidiosis is frequently accompanied by secondary bacterial infection [71, 105, 106, 112].

Prebiotics refer to nondigestible feed ingredients that promote the growth of probiotics and their activities in guts [113]. As illustrated in Table 4, the most common prebiotics, used in poultry, include inulin, arabinoxylooligosaccharides (AOS), fructooligosaccharides (FOS), mannan-oligosaccharides (MOS), xylooligosaccharides (XOS), isomaltooligosaccharides (IMOS), soy oligosaccharides (SOS), and pyrodextrins [92, 114]. These oligosaccharides are derived from the plants such as chicory, onion, garlic, asparagus, artichoke, leek, bananas, tomatoes, and wheat [92]. Dietary supplementation of these prebiotics to chicken feed has enhanced immune defence against pathogen infection and reduced the mortality rate [91, 115]. The mechanism of prebiotics is yet to be revealed, but they selectively stimulate beneficial bacteria in the intestinal system of the bird. The increasing number of beneficial microbiota excludes the harmful pathogens from colonization in the intestinal track of the bird. Subsequently, healthy hosts can produce a wide variety of bacterions and other immunomodulators that can stimulate macrophages to neutralize the pathogens [114]. Thus, prebiotic-mediated immunological changes may be partially due to direct interaction between prebiotics and gut immune cells as well as due to an indirect action of prebiotics via preferential colonization of probiotics and their products that interact with immune cells [85]. Therefore, prebiotics exert their functions mainly via increasing gut probiotics to suppress pathogens and boosting immune response in chickens to constrain gut pathogens [91]. Moreover, Bozkurt et al. reported that prebiotics diminished coccidial infection in chickens but kept marginal oocyst production that might serve as a source of live vaccine for uninfected chickens [116]. Two other publications emphasized that probiotics composed of* Bifidobacterium animalis* and* Lactobacillus salivarius* alleviated the detrimental impact of the* Eimeria* infection on chickens and improved growth performance [110, 111]. These findings suggest that prebiotics suppress coccidiosis plausibly via indirect regulation of increased probiotics and host immunity. Apparently, prebiotics share many similar anticoccidial mechanisms with probiotics. Overall, dietary supplementation of prebiotics is emerging as a novel approach to control coccidiosis.

### 2.4. Plants and Compounds with Multiple Mechanisms to Inhibit Coccidiosis

#### 2.4.1. Curcumin and* Curcuma longa*

As described in Table 2, turmeric (*C. longa*) has long been used as a spice and medicinal herb. One publication stated that* C. longa* showed anticoccidial activity [67, 117]. Another reported that curcumin (diferuloylmethane), an active compound in* C. longa*, consistently destroyed sporozoites of* E. tenella* [118]. Similarly, a combination of* A. annua* and* C. longa* showed anticoccidial efficacy in broilers challenged with a mixture of* E. acervulina* and* E. maxima* [119]. In addition, curcumin was shown to enhance coccidiosis resistance as evidenced by increased BW gains and reduced oocyst shedding and gut lesions [21, 67]. Consistently, curcumin elevated host humoral immunity to* Eimeria* species and diminished gut damage in poultry [21, 67].

#### 2.4.2. Polyacetylenes and* Bidens pilosa*

As described in Table 2, Yang et al. demonstrated that* B. pilosa* has exhibited anticoccidial activity in chickens infected with* E. tenella* as evidenced by survival rate, fecal oocyst count, gut pathology, body weight, and bloody stool [9]. Although the active compounds in* B. pilosa* responsible for anticoccidial action are unknown, this plant is a rich source of phytochemicals, such as 70 aliphatics, 60 flavonoids, 25 terpenoids, 19 phenylpropanoids, 13 aromatics, 8 porphyrins, and 6 other compounds [120]. Interestingly, one polyacetylene (1-phenyl-1,3-diyn-5-en-7-ol-acetate) and one flavonoid (quercetin-3,3-dimethoxy-7-0-rhamnoglucopyranose) in this plant have been proposed to be active compounds against the protozoan parasite,* Plasmodium* [121].

However, the identity of the active compounds needs to be further ascertained. The mechanism of* B. pilosa* and its active compounds is not clear. It is possible that this plant and its active compounds intervene in the initial phases of the* Eimeria* life cycle because the phases may be liable to chemical attack when compared with the oocyst whose wall is very resistant to physical and chemical insults. In addition,* B. pilosa* was shown to modulate host immunity [122], which might have impact on coccidiosis.

Compared to anticoccidial drugs,* B. pilosa* was shown to produce little or no drug resistance in* Eimeria* [9]. Botanicals developed low resistance in* Eimeria* probably because different compounds target multiple pathways related to drug resistance [9, 12].

It should be noted that the plant- and phytochemical-based remedies can be used* per se* or in combination with other anticoccidial agents. This idea was further confirmed by one publication indicating that* Echinacea*, an immunotherapeutic herbal extract, was used to boost the immunization efficacy in chickens in combination with anticoccidial vaccines [78].

## 3. Conclusions and Perspectives

Coccidiosis is a deadly and debilitating infectious disease in poultry, which is caused by enteric protozoan parasites,* Eimeria species*, in the guts of birds. These parasites damage the guts of the birds, leading to moderate clinical symptoms such as sick bird appearance, bloody stool, hemorrhage, and gut lesions and death. Pathogens (*Eimeria* species, swallowed oocyst counts, etc.), host genetics, and environmental factors could influence the clinical outcome of avian coccidiosis. Current prophylaxis and therapy for coccidiosis comprise anticoccidial chemicals, vaccines, and natural products. Plants are a rich source of phytochemicals against coccidiosis. Here, we summarized the chemistry and biology of over 68 plants and compounds which prevented and treated avian coccidiosis via the regulation of the life cycle of* Eimeria* species and host immunity and gut microflora in experimental and field trail studies. Emphasis was placed on recent advances in the understanding of the potential of these plants and compounds to prevent and/or treat avian coccidiosis. Moreover, the actions, mechanisms, and therapeutic potential of these plant compounds and/or extracts in avian coccidiosis and new insights into the advantage of plant extracts and compounds that simultaneously regulate* Eimeria*, bacteria, and immune cells were discussed. Comprehensive information about the structure, activity, and modes of action of these compounds can aid research and development of anticoccidial remedies.

## Figures and Tables

**Table 1:** Anticoccidial properties of plants.

S. number	Plant species (usage)	Usage^*∗*^	*Eimeria* species	Parameter measured	Reference
1	*S. flavescens*	Decoction	Et	WG ↑, OC ↓, BD ↓, LS ↓, and M ↓	[41]
2	*S. acutum*	Decoction	Et	WG ↑, OC ↓, BD ↓, LS ↓, and M ↓	[41]
3	*Q. indica*	Decoction	Et	WG ↑ and M ↓	[41]
4	*P. koreana*	Decoction	Et	WG ↑ and LS ↓	[41]
5	*U. macrocarpa*	Decoction	Et	LS ↓	[41]
6	*A. asiatica*	Decoction	Et	WG ↑ and LS ↓	[41]
7	*G. japonica*	Decoction	Et	LS ↓	[41]
8	*M. azedarach*	Decoction	Et	WG ↑ and LS ↓	[41]
9, 10	*P. nigrum *and* U. dioica*	Ethanolic extract	Mixed species	OC ↓	[42]
11	*A. sieberi*	Petroleum ether extract	Et	OC ↓, BD ↓, LS ↓, and M ↓	[43–45]
12	*L. sativum*	Ethanolic extract	Et	OC ↓, LS ↓, M ↓, and WG ↑	[46]
13	*F. vulgare*	Ground leaves powder	Et	OC ↓, LS ↓, M ↓, WG ↑, and BD ↓	[47]
14	*M. lucida*	Ground leaves powder	Mixed species	WG ↑ and OC ↓	[48]
15	*C. swynnertonii*	Ethanolic resinous extract	Oocysts	OC ↓, M ↓, and WG ↑	[49]
16	*M. oleifera*	Acetone leaves extract	Mixed species	WG ↑ and OC ↓	[50]
17	*Origanum *spp.	Essential oil	Mixed species	OC ↓, LS ↓, M ↓, and WG ↑	[51]
18	*L. nobilis*	Essential oil	Mixed species	OC ↓, LS ↓, M ↓, and WG ↑	[51]
19	*L. stoechas*	Essential oil	Mixed species	OC ↓, LS ↓, M ↓, and WG ↑	[51]
20	*M. paradisiaca*	Methanolic extract	Et	OC ↓ and PCV ↑	[52]
21	*M. stenopetala*	Ground leaves powder	Et	OC ↓, LS ↓, and WG ↑	[53]
22	*S. nigrum*	Decoction	Et	WG ↑ and FC ↑	[54]
23	*M. arvensis*	Decoction	Et	WG ↑ and FC ↑	[54]
24	*M. indica*	Decoction	Et	WG ↑ and FC ↑	[54]
25	*M. azedarach*	Fresh juice	Mixed species	OC ↓	[55]
26	*T. violacea*	Acetone extract	Et	OC ↓, LS ↓, and FC ↑	[56]
27	*V. vinifera*	Acetone extract	Et	OC ↓, LS ↓, and FC ↑	[56]
28	*A. afra*	Acetone extract	Et	OC ↓, LS ↓, and FC ↑	[56]
29	*G. rhois*	Ground powder	Et	OC ↓, LS ↓, M ↓, and WG ↑	[57]
30, 31, 32	*Q. infectoria*, *R. chinensis*, and* T. chebula*	?	Et, Em, and Ea	OC ↓, LS ↓, and M ↓	[7]

Et: *E. tenella*; Ea: *E. acervulina*; Ema: *E. maxima*; Eb: *E. brunetti*; En: *E. necatrix*; Emi: *E. mivati*; WG: body weight gain; OC: oocyst count; FC: feed consumption; M: mortality; EO: essential oil; BD: bloody diarrhea; FCR: feed conversion ratio; LS: lesion scores; ↑: improvement/increase/higher; ↓: decrease/lower; PCV: packed cell volume; *∗*: whole plants and/or aerial parts of plants were used for tests unless indicated otherwise; ?: unknown.

**Table 2:** Phytochemicals interfering with the life cycle of *Eimeria* species.

S. number	Compound	Structure and formula	Plant	Mechanism	*Eimeria* species	Reference
1	Artemisinin	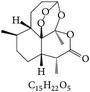	*A. annua*	Inhibition of oocyst wall formation and sporulation via oxidative stress	Et, Ea, and Ema	[20–23, 58]
2	Tannin	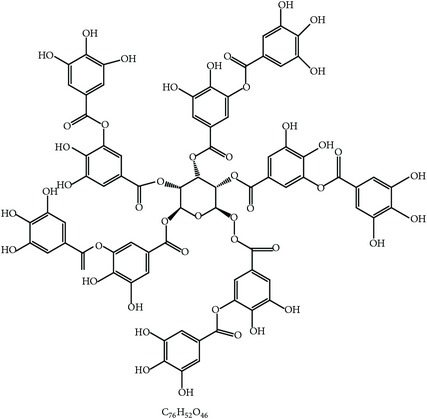	*P. radiata*	Inhibition of sporulation	Et, Ea, and Ema	[25]
3	Allicin	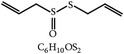	*A. sativum*	Inhibition of sporozoites	Et	[26, 28]
4	Polyacetylene	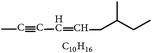	*B. pilosa*	Inhibition of sporozoites; immune modulation	Et	[8, 9]
5	Berberine	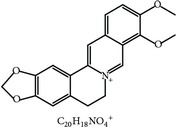	*A. lyceum*	Inhibition of sporozoites by oxidative stress	Et	[59]
6	N-3 fatty acids	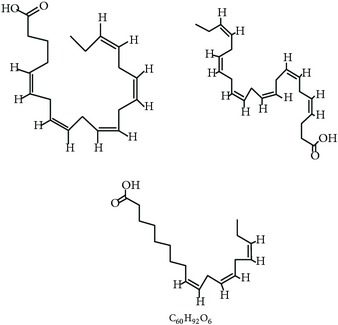	*L. usitatissimum*	Oxidative stress	Et	[21]
7	Flavonoid	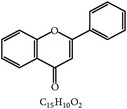	*A. conyzoides*	Oxidative stress	Et	[31]
8	Vernoside	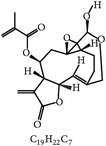	*V. amygdalina*	Oxidative stress	Et	[32]
9	Papain	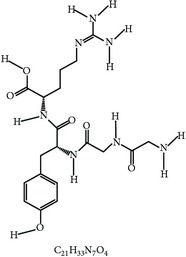	*C. papaya*	Digestion of sporozoites in caeca	Et	[32]
10	Betaine	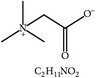	*O. vulgare*	Stabilizing intestinal structure and function	Et, Ea, and Ema	[25, 36]
11	Essential oil (carvacrol, thymol, and terpinene-*γ*)	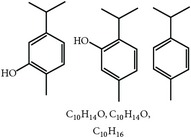	*O. compactum*	Destruction of sporozoites	*Eimeria* species	[12, 60]
12	Essential oil (*β*-thujone, 1,8-cineol, and p-cymene)	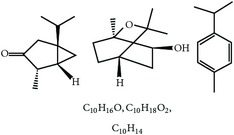	*A. absinthium*	Prevention of oocyst development	*Eimeria* species	[61, 62]
13	Essential oil (cineol, *α*-pinene, and bornyl acetate)	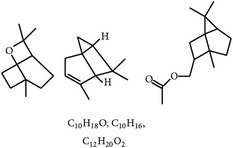	*R. officinalis*	Antioxidant and destruction of oocysts	*Eimeria* oocysts	[61, 63, 64]
14	Saponin	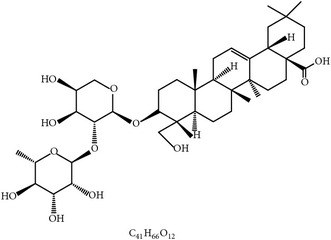	*M. cordifolia*, *M. citrifolia*, *M. arboreus*, and *C. tetragonoloba*	Destruction of oocysts and parasites	Et	[35]
15	Essential oil (eugenol and eugenyl acetate)	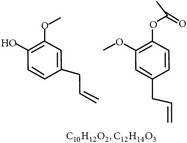	*S. aromaticum*	Destruction of oocysts	Et	[51, 61]
16	Essential oil (terpinen-4-ol and terpinene-*γ*)	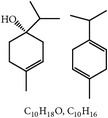	*M. alternifolia*	Destruction of oocysts	*Eimeria* oocysts	[61, 65]
17	Essential oil (limonene and linalool)	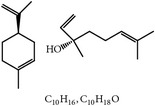	*C. sinensis*	Destruction of oocysts	*Eimeria* oocysts	[61, 66]
18	Essential oil (thymol and p-cymene)	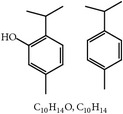	*T. vulgaris*	Destruction of oocysts	*Eimeria* oocysts	[12, 61]
19	Curcumin (diferuloylmethane)	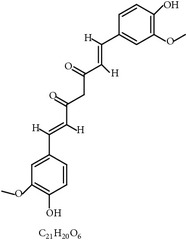	*C. longa*	Inhibition of sporozoites; immune modulation	Et and Ema	[21, 67]
20	Maslinic acid	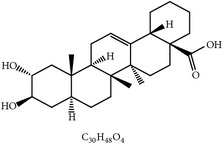	*O. europaea*	?	Et	[37]
21	Proanthocyanidin	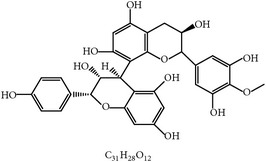	Grape seed	Antioxidant	Et	[38]
22	Selenium	?	*C. sinensis*	Inhibition of sporulation	Et, Ea, and Ema	[30]
23	Febrifugine	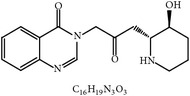	*D. febrifuga*	Inhibition of multiplication	Et	[10, 39, 68]

Et: *E. tenella*; Ea: *E. acervulina*; Ema: *E. maxima*; Eb: *E. brunetti*; En: *E. necatrix*; Emi: *E. mivati*; ?: unknown.

**Table 3 tab3:** Phytochemicals regulating host immunity against *Eimeria* species.

S. number	Compound	Structure and formula	Plant	Mechanism	*Eimeria* species	Reference
24	Arabinoxylans	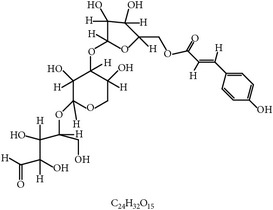	*T. aestivum*	Immune stimulation	Et, Ea, Ema, and En	[69]
25	Cinnamaldehyde	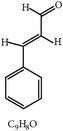	*C. cassia*	Immune modulation	Et, Ea, and Ema	[70, 71]
26	Acemannan	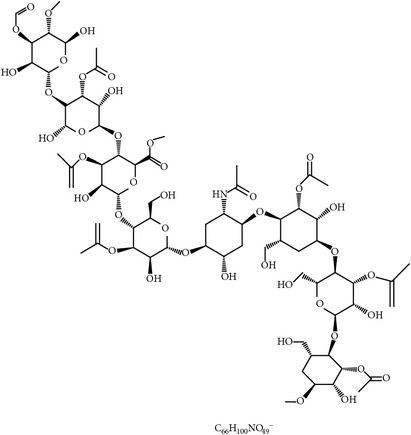	*A. vera*	Immune stimulation	*Eimeria *spp.	[72, 73]
27	Lectin	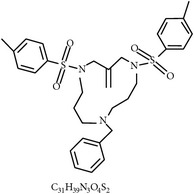	*F. fraxinea*	Immune stimulation	Ea	[74]
28	Propyl thiosulfinate	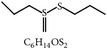	*A. sativum*	Protective immunity	Ea	[75, 76]
29	Tannin (pedunculagin)	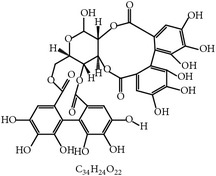	*A. officinalis*	Immune stimulation	*Eimeria *spp.	[77]
30	Chicoric acid	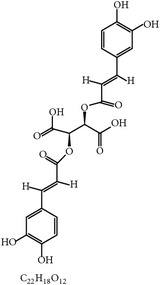	*E. purpurea*	Immune stimulation	*Eimeria *spp.	[78, 79]
31	Mushroom polysaccharide	?	*L. edodes *and *T. fuciformis*	Immune stimulation	Et	[80]
32	Phenolics compounds	?	*P. salicina*	Immune stimulation	Ea	[81, 82]

Et: *E. tenella*; Ea: *E. acervulina*; Ema: *E. maxima*; Eb: *E. brunetti*; En: *E. necatrix*; ?: unknown.

**Table 4:** Phytochemicals with prebiotic function for gut microbiota.

S. number	Prebiotics	Chemical structure	Plant	Effects on poultry microbiota	Reference
33	Inulin	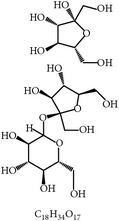	Chicory	Enhancing gut microflora, morphology, and immunity	[83, 84]
34	Mannan-oligosaccharides	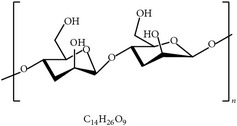	Fungi and yeast	Increasing digestion and gut microbiota	[85, 86]
35	Xylooligosaccharides	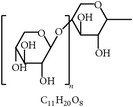	Bamboo shoots, fruits, vegetables, and wheat bran	Increasing *Lactobacillus* in colon	[87]
36	Isomaltooligosaccharides	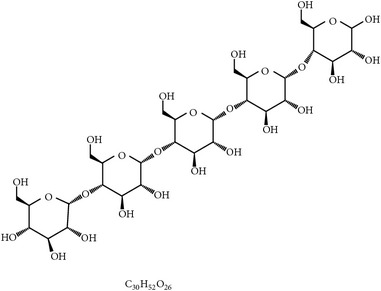	Starch	Increasing cecal probiotics and fatty acids	[88, 89]
37	Soy oligosaccharides	?	Soybean	Changing microbiota	[90]
38	Pyrodextrins	?	Sucrose	Increasing gut microbiota and growth performance	[91]
39	Oligofructose	?	Asparagus, sugar beet, garlic, onion, chicory, and artichoke	Increasing digestion and gut microbiota	[85, 86, 92]
40	Arabinoxylooligosaccharides	?	Wheat bran	Increasing digestion and gut microbiota	[93–95]

?: unknown.
